# Data Processing and Information Classification—An In-Memory Approach

**DOI:** 10.3390/s20061681

**Published:** 2020-03-18

**Authors:** Milena Andrighetti, Giovanna Turvani, Giulia Santoro, Marco Vacca, Andrea Marchesin, Fabrizio Ottati, Massimo Ruo Roch, Mariagrazia Graziano, Maurizio Zamboni

**Affiliations:** 1 Department of Electronics and Telecommunication (DET), Politecnico di Torino, Corso Castelfidardo 39, 10129 Torino, Italy; milena.andrighetti@studenti.polito.it (M.A.); giulia.santoro@polito.it (G.S.); marco.vacca@polito.it (M.V.); andrea.marchesin@studenti.polito.it (A.M.); fabrizio.ottati@studenti.polito.it (F.O.); massimo.ruoroch@polito.it (M.R.R.); maurizio.zamboni@polito.it (M.Z.); 2 Department of Applied Science and Technology (DISAT), Politecnico di Torino, Corso Castelfidardo 39, 10129 Torino, Italy; mariagrazia.graziano@polito.it

**Keywords:** bitmap indexing, processing in memory, memory wall, big data, internet of things

## Abstract

To live in the information society means to be surrounded by billions of electronic devices full of sensors that constantly acquire data. This enormous amount of data must be processed and classified. A solution commonly adopted is to send these data to server farms to be remotely elaborated. The drawback is a huge battery drain due to high amount of information that must be exchanged. To compensate this problem data must be processed locally, near the sensor itself. But this solution requires huge computational capabilities. While microprocessors, even mobile ones, nowadays have enough computational power, their performance are severely limited by the Memory Wall problem. Memories are too slow, so microprocessors cannot fetch enough data from them, greatly limiting their performance. A solution is the Processing-In-Memory (PIM) approach. New memories are designed that can elaborate data inside them eliminating the Memory Wall problem. In this work we present an example of such a system, using as a case of study the Bitmap Indexing algorithm. Such algorithm is used to classify data coming from many sources in parallel. We propose a hardware accelerator designed around the Processing-In-Memory approach, that is capable of implementing this algorithm and that can also be reconfigured to do other tasks or to work as standard memory. The architecture has been synthesized using CMOS technology. The results that we have obtained highlights that, not only it is possible to process and classify huge amount of data locally, but also that it is possible to obtain this result with a very low power consumption.

## 1. Introduction

Nowadays many applications used everyday, defined as data-intensive, require a lot of data to process. Examples are the databases manipulation and image processing. This requirement is the effect of the fast improvement of CMOS technology, that has lead to the creation of very powerful and flexible portable devices. These devices are full of sensors that continuously acquire data. Data can be elaborated remotely by powerful servers, but sending a lot of information through electromagnetic waves requires a huge amount of energy, severely impacting the battery life of mobile devices. The only solution is to elaborate data locally, on the mobile device itself.

Thanks to the scaling of transistors size, mobile microprocessors are now theoretically capable of such computation. Unfortunately, memory scaling has been following a different path, resulting still in slow accesses compared to processors computing speed. This discrepancy in performance harms the computing abilities of the CPU, since the memory cannot provide data as quickly as required by the CPU. This problem is called *Von Neumann bottleneck* or *Memory Wall*. The idea that took form to solve this problem is to null the distance between processor and memory, removing the cost of data transfer and create a unit which is capable of storing information and of performing operation on them. This idea takes the name of Processing-in-Memory.

Many in literature have approached the “in-memory” idea. Some narrowing the physical distance between memory and computation unit by creating and stacking different layers together. But even if the two units are moved very close to each other, they are still distinct components. Others exploited intrinsic functionality of the memory array or slightly modified peripheral circuitry to perform computation.

Among the many example provided by literature, one of the best fitting representative of the PIM concept is presented in Reference [1]. In this work the proposed architecture is a memory array in which the cell itself is capable of performing logical operations aimed at solving Convolutional Neural Networks (CNN). In this paper, our main goal is to introduce a proper example of Processing-in-Memory, choosing Bitmap Indexing as an application around which the architecture is shaped. In the design, it was not used a specific memory technology because the idea is to provide a worst-case estimation and it was also meant to leave space for future exploration to implement the cell with a custom model of the memory cell. The Bitmap Indexig algorithm has been chosen because it is used for data classification. This is one of the most important task that must be performed by such mobile devices. Being able to classify data allows to understand which data must be sent to remote servers and which not, greatly reducing the overall power consumption. The presented architecture is a memory array in which each cell is both capable of storing information and to perform simple logical operation on them. A characteristic of our architecture is its modularity. The architecture is divided in independent memory banks. A memory bank can work both on its own or interacting with other banks. Moreover it is possible to build the array with as many banks as needed. This feature lead to great flexibility and high degree of parallelism. The structure was eventually synthesized for analysis purposes, in a 8.5 KB square array, using CMOS 45 nm and 28 nm. The storage segment of the proposed PIM cell was synthesized as a latch. The evaluation showed great results, achieving a maximum throughput of 2.45 Gop/s and 9.2 Gop/s respectively for the two technologies used. This paper is the extended version of our prior work [2]. In the conference paper the general idea was introduced. Here we greatly expand the architecture, moving from the idea to the real implementation. The novelty of this work, in comparison with other works presented in the literature, consists in an enhanced architecture characterized by a high level of granularity and flexibility.

## 2. Background

The Processing-in-Memory paradigm was born to solve the *Von Neumann bottleneck*, which is characterized by the gap in performance between memory and processor. Processing-in-Memory thus tries to reduce the disparity by merging together storage and processing units. Processing-in-Memory (PIM) can be approached in different ways, depending on the architecture or the technologies to use. A lot of examples can be found in literature, some of them will be depicted in the following, grouped in categories.

### 2.1. Magnet-Based

*Magnetic Random Access Memory* (MRAM) is a non-volatile memory that uses Magneto-Tunnel Junctions as its basic storage element. Thanks to their dual storage-logic properties, MTJs are suitable to implement hybrid logic circuits with CMOS technology suited to implement the PIM principle. In Reference [3] is presented a MTJ-CMOS Full Adder, which compared to a standard only-CMOS solution showed better results. In Reference [4] the authors proposed an MTJ-based TCAM, in which the logic part and the storage element are merged together, and an MTJ-based Non-Volatile FPGA exploiting MTJs and combinatorial blocks. Both structures resulted in a more compact solution with respect to conventional ones.

In Reference [5] it is proposed a different way to implement Nano Magnetic Logic (NML) exploiting the MRAM structure. Since the basic concept of the NML technology is the transmission of information through magnetodynamic interaction between neighbouring magnets, the MRAM structure has been modified so that MTJs could interact with each other. Another example is represented by PISOTM [6], an architecture based on SOT-RAM. It is a reconfigurable architecture in which the main advantage is that the storage and logic element result identical and for this reason technology conflict is avoided.

### 2.2. 3D-Stacking

According to the 3D-Stacking approach multiple layers of DRAM memory are stacked together with a logic layer that can be application-specific ([7,8]) or general purpose [9]. In Reference [7] the XNOR-POP architecture was designed to accelerate CNNs for mobile devices. It is composed of Wide-IO2 DRAM memory with the logic layer modified according to the XNOR-Net requirements. In Reference [8] it is proposed an architecture for data intensive applications, where a PIM layer made of memory and application-specific logic is sandwiched between DRAM dies connected together using TSVs. An example of general purpose 3D-stacking is 3D-MAPS in Reference [9]. A multi-core structure is used, and every core is composed of a memory layer and a computing layer.

### 2.3. ReRAM-Based

*Resistive RAM* is a non-volatile memory that uses a metal-insulator-metal element as storage component. The information is represented by the resistance of the device that can be either high (HRS) or low (LRS). To switch between states the appropriate voltage has to be applied to the cell. The common structure of a ReRAM array is a crossbar, a structure used in matrix-vector multiplication, commonly found in neural networks applications. PRIME [10], an architecture aimed at accelerating Artificial Neural Networks is an example of this kind of implementations. PRIME is compliant with the in-memory principle, since the computation is performed directly into the memory array with few modifications to the peripheral circuitry. Memory banks are divided intro three sub-arrays each with a specific role in the architecture. In Reference [11] is proposed a 3D-ReCAM based architecture to accelerate the BLAST algorithm for DNA sequence alignment. The architecture, named RADAR, aims to move the operations in memory, this way there is no need to transfer the DNA database. In Reference [12] is presented a non-volatile intelligent processor built on a 150 nm CMOS process with HfO RRAM. The structure is capable of both general computing and the acceleration of neural networks, in fact it is provided with a FCNN Turbo Unit, enhanced with low-power MVM engines to perform FCNN tasks.

Another application that is limited by the Memory Wall problem is Graph Processing. In Reference [13] is proposed a ReRAM-based in-memory architecture as a possible solution. The structure is composed of multiple ReRAM banks, divided into 2 types: graph banks that are used to map the graph and to store its adjacency list and a master bank which stores metadata of the graph banks. This allows to process the graphs that are stored inside the memory. In Reference [14] is presented PLiM, a programmable system composed of a PIM controller and a multi-bank ReRAM which can work both as a standard memory and as a computational unit, according to the controller signals. PLiM implemented only serial operation to keep the controller as simple as possible. In Reference [15] the authors presented ReVAMP, an architecture composed of two ReRAM crossbars, supporting parallel computations and VLIW-like instructions. To perform logic operations ReVAMP exploits the native properties of ReRAM cells that implement a majority voting logic function.

### 2.4. PIM

In Reference [16] the authors presented TOP-PIM, a system composed of an host processor surrounded by several units characterized by 3D-stacked memories with an in-memory processor embedded on the logic die. In Reference [17] is proposed DIVA, a system in which multiple PIM chips serve as smart-memory co-processors to a standard microprocessor aimed at improving bandwidth performance for data intensive applications executing computation directly in memory and enabling a dedicated communication line between the PIM chips. In Reference [18] is presented Terasys, a massively parallel PIM array. The goal of Terasys was to embed an SIMD PIM array very close to an host processor in order for it to be seen both as a processor array and conventional memory. As solution for large-scale graph processing performance bottleneck, in Reference [19] the authors proposed Tesseract, a PIM architecture used as an accelerator for an host processor. Each element of Tesseract has a single-issue in-order core to execute operations, moreover, the host processor has access to the entire Tesseract’s memory whilst each core of Tesseract can interact only with its own. Tesseract does not depend on a particular memory organization, but it was analyzed exploiting Hybrid Memory Cube (HMC) as baseline. Such a structure proved to perform better than traditional approaches thanks to the fact that Tesseract was able to use more of the available bandwidth. In Reference [20] is presented Prometheus, a PIM-based framework, which proposes the approach of distributing data across different vaults in HMC-based systems with the purpose of reducing energy consumption, improving performance and exploiting the high intra-vault memory bandwidth.

In Reference [21] is proposed a solution to accelerate Bulk Bitwise Operations. PINATUBO is an architecture based on resistive cell memories, such as ReRAMs. The structure is composed of multiple banks which are also subdivided into mats. Pinatubo is able to eliminate the movement of data, since computation is performed directly inside memory, executing operations between banks, mats and subarrays. This way PINATUBO interacts with CPU only for row addresses and control commands. Another example of PIM architecture to accelerate bulk bitwise operations was conceived by the authors of Reference [22], who presented Ambit, an in-memory accelerator which exploits DRAM technology to achieve total usage of the available bandwidth. The DRAM array is slightly modified to perform AND, OR and NOT operations. Moreover, the CPU can access Ambit directly, this way it is not necessary to transfer data between CPU memory and the accelerator. In Reference [23] is proposed APIM, an Approximate Processing-in-Memory architecture which aims to achieve better performance despite a decrease in accuracy. It is based on emerging non-volatile memories, such as ReRAM and it is composed of a cross-bar structure grouped in blocks. All the blocks are structurally identical but divided into data and processing blocks. They are linked together through configurable interconnections. Furthermore APIM is able to configure computation precision dynamically, so that it is possible to tune the accuracy runtime.

In Reference [24] is presented ApproxPIM, an HMC-based system in which each vault is independent from one another and communication with the host processor is based on a parcel transmission protocol. This results in energy and speedup improvements with respect to the used baselines. In Reference [25] the authors presented MISK, a proposal to reduce the gap between memory and processor. Since data movement imply a great energy cost, MISK is intended to reduce it by implementing a monolithic structure, avoiding physical separation between memory and CPU. In fact, MISK is to be integrated into the cache and it is not conceived to work on its own, but embedded in the CPU. This way it is possible to achieve great results in terms of energy-per-cycle and execution time. In Reference [26] is introduced Gilgamesh, a system based on distributed and shared memory. It is characterized by a multitude of chips, called MIND chips, which are connected together through a global interconnection network. Each chip is a general purpose unit equipped with multiple DRAM bank and processing logic. In Reference [27] Smart Memory Cube is presented, a PIM processor built near the memory, in particular HMC, which is connected to an host processor. HMC vault controls are modified to perform atomic operations. The PIM processor interacts with the host processor so that smaller tasks are executed directly side by side the memory.

In References [28,29], the authors presented in-memory architectures on which the Advanced Encryption Standard (AES) algorithm was mapped, showing great result in speed and energy saving compared to other solutions. In Reference [1], the authors presented an architecture based on the in-memory paradigm aimed at Convolutional Neural Networks (CNN). The structure is a memory array in which each cell is provided with both storage and computation properties and with the support of an additional weight memory which is designed to support CNN data flow and computation inside the array. This structure showed great result compared with a conventional CNN accelerator in terms of memory accesses and clock cycles.

## 3. The Algorithm

The Processing-in-Memory principle requires that the storage and logic components are merged together. In order to implement an architecture compliant with such a requirement it was necessary to firstly shape it according to a suitable application. For this purpose Bitmap indexing was selected. Bitmap indexes are often used in database management systems.

**Figure 1:**
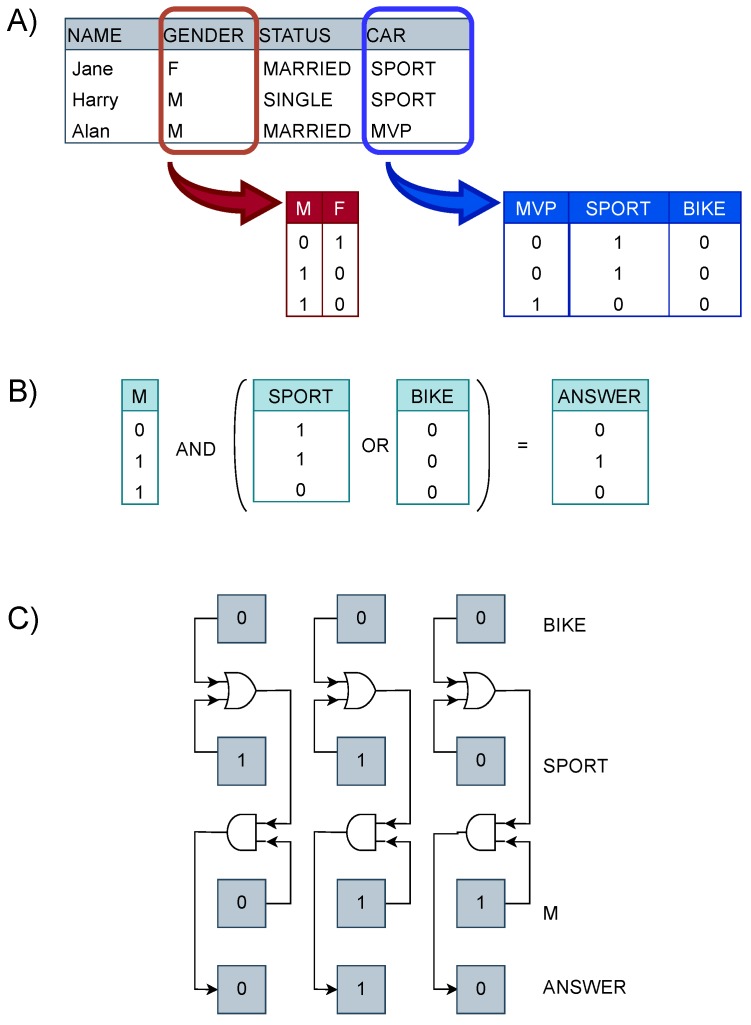
(**A**) Given a table, bitmap indexing transforms each column in as many bitmap as the number of possible key-values for that column (**B**) In order to answer a query logic bitwise operations are to be performed (**C**) Practical scheme of the execution of the query.

Taking as an example the simple database in Figure 1A, each column of the database represents a particular characteristic of the profile of the entry described in one row. Suppose a search on the database is to be performed to create a statistic on how many men possess a sport car or a motorbike. Such a query would imply looking for all the men and then excluding the ones that do not own the specified vehicles. If the database is big this operation would require a long response time. Bitmap indexing was introduced to solve this issue. Bitmap indexing transforms each column of a table in as many indexes as the number of distinct key-values that particular column can have.

A bitmap index is a bit array in which the *i*-th bit is set to 1 if the value in the *i*-th row of the column is equal to the value represented by the index, otherwise it is set to 0 (Figure 1A). Thus, bitmap indexing allows to fragment search queries in simple logic bitwise operations (Figure 1B). This way it is not necessary to analyze the whole database discarding unwanted data, but only to operate on selected indexes. Bitmap indexing can provide great results in response time and in storage requirements since it can be compressed. Bitmap indexing is suited for entries with a number of possible values smaller than the depth of the whole table. This technique is mostly functional for queries regarding the identification of the position of specific features, for this reason to answer an “how many” query it is necessary to insert a component that counts the hits obtained. Summing up, a query can be decomposed in simple logic operations which are performed between indexes, processing bits belonging to the same position in the array (Figure 1C).

**Figure 2:**
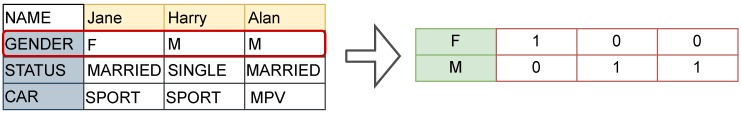
Column-oriented memory organization.

Clearly, Bitmap indexing results compatible with the Processing-in-Memory paradigm, since it is characterized by simple logic bitwise operations and its data format make it easy to embed in memory. However, bitmap indexing involves operations between columns of a table. If we consider memory organization and imagine to maintain the column-row distribution of the table in memory, this would imply to access multiple rows and then discard all the data that do not belong to the desired indexes. This approach would be too costly. For this reason for our implementation a column-oriented was preferred, which means that the entire table is stored transposed, so that now, applying bitmap indexing, indexes lie on rows (Figure 2).

Thanks to this method, to access an index it is only necessary to access a row and consequently operations between indexes result in operations between memory rows. In this implementation we thus consider the indexes distributed on rows in a memory array. We also take into account two types of query, *simple* and *composed*. A simple query is composed of only one operation (e.g., “Who is female and married?”) whilst a composed one is characterized by intertwined operations (e.g., Figure 1B). Considering the composed query depicted in Figure 1B the operations to perform would be:

1. Access the first operand;
2. Access the second operand;
3. Execute bitwise operation between the two operands;
4. Read result;
5. Execute bitwise operation between computed result and third index;
6. Count the hits obtained;
7. Read final result;

While to answer a simple query only steps 1–4 are needed. The goal is then to implement the just introduced algorithm directly inside a memory array.

## 4. The Architecture

The architecture proposed in this paper present a possible solution for the Von Neumann bottleneck implementing a proper *in-memory* architecture, where logic functions are implemented directly inside each memory cell, in contrast with the *near-memory* approach seen in some state-of-the-art implementations, where logic operations are performed with logic circuits located on the border of the memory array. Moreover, this architecture was intended to overcome the limits provided by specific technologies by keeping the development of the architecture technology-independent, in order to implement a configurable architecture with the highest degree of parallelism achievable.

A memory array is composed of many storage units, each of which is made of multiple memory cells. Cells are the basic element of the memory itself. Therefore, in order to implement an entire memory array aimed at executing the Bitmap indexing algorithm, firstly it is necessary to define the structure of the memory cell.

**Figure 3:**
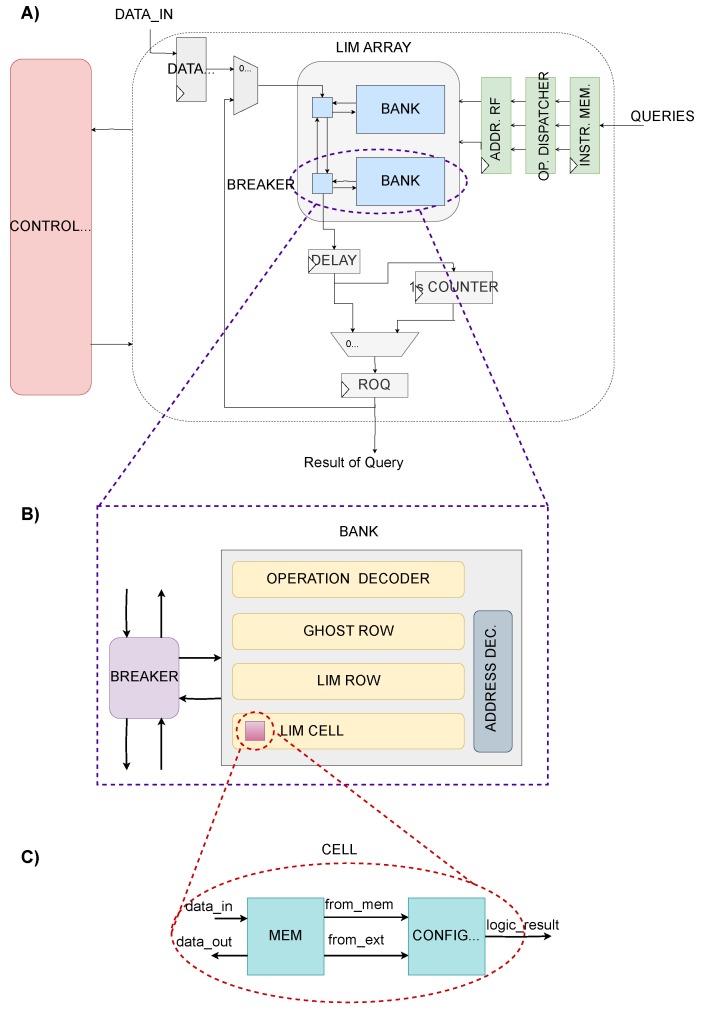
(**A**) Overview of the complete architecture. (**B**) Structure of the duo Bank-Breaker. (**C**) Insight of the Processing-In-Memory (PIM) cell.

According to the specifications required by the Bitmap indexing, the cell has to be able to perform simple logic operations interacting with other cells in the array. This means that our cell should have both storage and logic properties. Indeed, the basic cell of the PIM array is provided with an element that store information and a configurable logic element which performs AND, OR, XOR operations with all the combinations of input (e.g., $A,\bar{A}$), between the stored information and the one coming from another cell (Figure 3). The system has indeed the granularity of a single bit, meaning that every memory cell executes a logic operation.

Other than standard memory features the PIM cell can interact with other cells, according to its control input. As every single cell in the array has the ability to perform computation, it is necessary to choose which cell will be executing the operation and which will be read. In order to implement it, the designated passive cell is read and the stored data travels to the operative cell. To avoid interference between inactive cells, the output lines of cells that are not used are interrupted. To implement the bitwise feature each cell of a row has its input and output line common to any other cell belonging to the same column of different rows.

In Figure 3, the whole structure is depicted. Noticeably, other than the array, the architecture is composed of a control unit and some additional components, such as the counter (for counting ones) and register files. Focusing on the array, like any standard memory, it was divided into multiple banks. Each bank is associated with a *breaker* that manages data flow from and to the bank. A bank represents the smallest degree of parallelism of the architecture. This means that in a bank it is possible to execute one operation at a time. The system has also a second level of granularity because thanks to the breakers every bank can work independently. This solution provides at the same time a high level of granularity and flexibility. Banks can execute operations between its rows or can work with other banks, making interact rows belonging to different banks, while other banks work on different operations in parallel. As a consequence, supposing each bank in the array works on a different operation by itself, the maximum degree of parallelism achievable is equal to the number of banks in the array. The *Bidirectional Breaker* is in charge of managing relations between its bank and the rest of the array. According to the control input, the breaker can be passive, that is, letting data pass through without disturbing its bank so that the bank can work on its own or be silent. The breaker can also be active and diverting data to or from its bank.

A bank is composed of multiple PIM rows and one *Ghost row* which is provided only with memory properties used to store temporary operation results. The Ghost row has the input line connected to the logic result output line of the PIM rows, whilst its output line is common with the PIM rows. This way it is possible to read the Ghost row or use its content for further computation. As in standard memories, each row is fragmented in multiple words. This means that operations are actually performed between words belonging to different rows. The result is then temporary saved in the Ghost word corresponding to the same word address of the word which executed the operation. This was implemented to avoid the need to manage a third address. To handle all the configuration signals needed to manage the correct execution, two decoders were needed inside each bank. One that sets the configuration for the logic operation to execute, sending it to the right row. The second was implemented to control addresses, data flow inside the bank and to distinguish between standard memory mode and PIM operation mode. Since a simple AND operation can be performed in one bank in a single clock cycle, imaging of having multiple banks definitely increase the number of operations that can be executed in one clock cycle in parallel. The same reasoning goes for a composed operation which takes two clock cycles. The throughput is directly proportional to the number of banks in the memory block. So, the larger the number of banks, the larger the memory block and also the larger the throughput.

In Figure 3, it is highlighted that, other than the array, there are some additional components which are used to guarantee the correct functioning of the entire structure.

The *Instruction Memory* is used to collect the queries to execute. It consists in a register file, having as many registers as the number of banks, with an input parallelism equal to the length of a complete query (i.e., two complete addresses and a logic operation configuration string). A composed query is treated as the combination of two distinct queries, which means that a composed query will occupy two consecutive registers of the Instruction Memory. Clearly, even if the architecture was configured to exploit its maximum potential by implementing the bitmap indexing algorithm, it can be configured to perform additional algorithms. For reconfigurability purposes the instruction memory had to be implemented as wide as possible, but most likely it will not be updated fully each time. In order to avoid conflicts the *Operation Dispatcher* is in charge of blocking any old query. Since a query can take place between any couple of addresses in the array, it is necessary to sent the addresses to their respective bank. The Operation Dispatcher thus reorders addresses and sends them to their own bank. After the correct reordering, to ensure synchronization the addresses are sampled by the *Address Register File* which loads the addresses and sends them to the array.

As illustrated previously, results of bitwise logic operations answer to queries in where clause. To count the number of ones (“1”) in the “how many” clause it was inserted a ones counter of logic “1” connected with the output of a delay register. The register was added to ensure timing constraints given by the counter. A simple counter that processes the data input bit-by-bit and increments by one for each “1” found was too slow. Therefore, a tree-structured counter was implemented. Firstly, the data array is fragmented into D segments, each of $\frac{N}{D}$-bits. All segments are then analyzed at the same time and the ones contained in each segment are counted. Finally, all the factors are added together to obtain the final sum. Also, all the adders that form the tree-structure are of the same dimension computed to avoid overflow.

**Figure 4:**
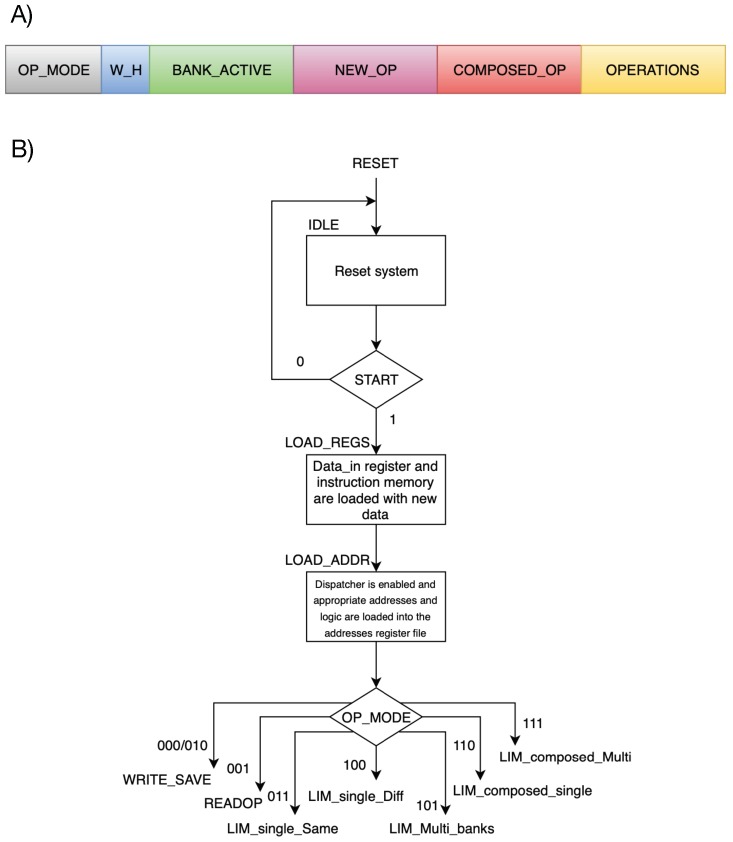
(**A**) Composition of a complete query. (**B**) Preliminary stages.

The architecture was conceived to incorporate as many features as possible and at the same time trying to keep the control circuits as simple as possible. The implemented structure is versatile and can work in 8 different operation modes, discerned among traditional memory operations and PIM operations based on the position of the two operands and the desired parallelism: (1) Write; (2) Read; (3) Save result; (4) PIM simple single bank; (5) PIM simple different banks; (6) PIM multiple banks; (7) PIM composed; (8) PIM multiple composed. Each operation mode is the starting point of a query, which is composed as shown in Figure 4A. The FSM chart of all operation modes are reported in Figure 4B.

The developed architecture is a modular configurable parallel architecture that implements the concept of Processing-in-Memory to perform bitwise logic operations directly inside the memory, making it suitable for other applications other than Bitmap indexing, as long as they are based on bitwise.

## 5. Results and Conclusions

The architecture was fully developed in VHDL (VHSIC Hardware Description Language). In order to evaluate its performance a 8.704 KB square memory array was analysed. The array distribution consisted in 16 banks with 16 bit data size. All the internal structures have been kept para- metric to give the possibility to implement the architecture composed of how many banks, rows and words needed according to the target database. From a MATLAB script (or from an external source in the case of the bitmap) were extracted both the bitmap and the queries to execute. The files were then set as input for the VHDL Testbench and finally it was run a simulation of the queries to feed the PIM architecture. When started, the script enters a loop that terminates only when the user decides not to create any more queries and a file generated as output. The completion of the query is assisted by two pop-up windows: one shows the internal composition of the memory and the other shows the available logic operations and their correspondent code.

**Figure 5:**
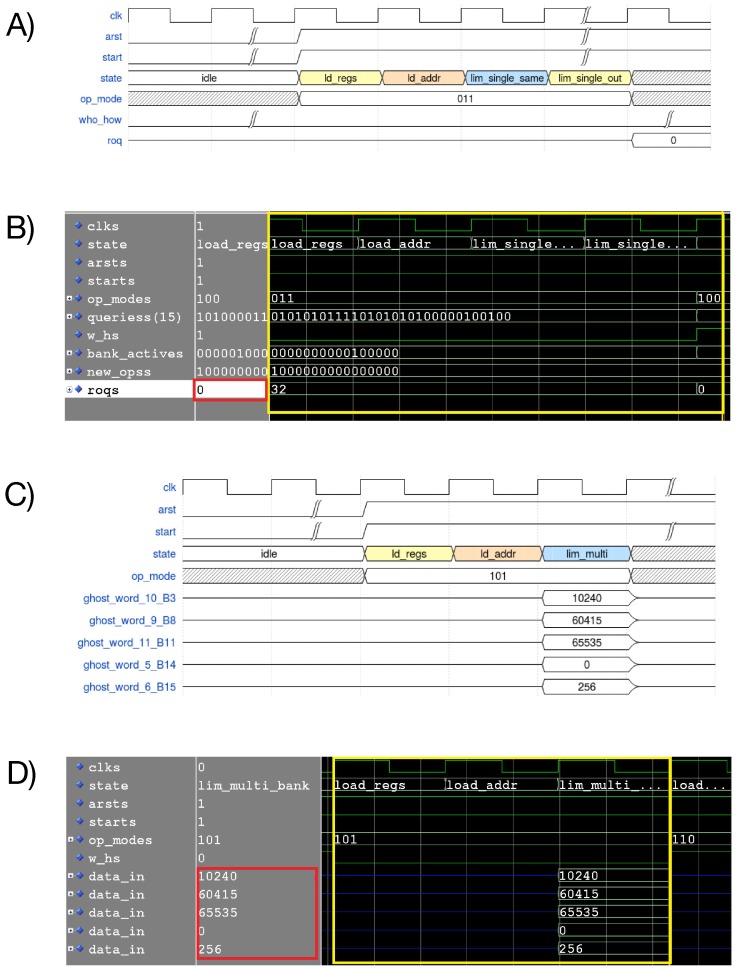
(**A**) Expected waveform of a LIM single same bank AND operation. (**B**) Waveform of a LIM single same bank AND operation. (**C**) Expected waveform of a PIM multiple operations. (**D**) Simulated waveform of a PIM multiple-bank operation.

All eight operation modes were tested with Modelsim to ensure the correct functioning. Two examples of operation mode are reported in Figure 5, it shows two examples of logic behavior (expected and simulated) of the proposed architecture.

The architecture was later synthesized with Synopsys Design Compiler using 45 nm BULK and 28 nm FDSOI CMOS technologies (Table 1). By using Synopsys Design Compiler latches and logic gates are used to implement the memory cell, so the results are not optimized as they will be if a custom transistor layout was created for the memory cell.

As the fundamental element of the whole structure, the Cell was analyzed and optimized. The obtained results are reported in Table 1 and Table 2.

From, Table 1 it is possible to evince the the area overhead is 55%. The overhead in terms of power dissipation is similar.

**Figure 6:**
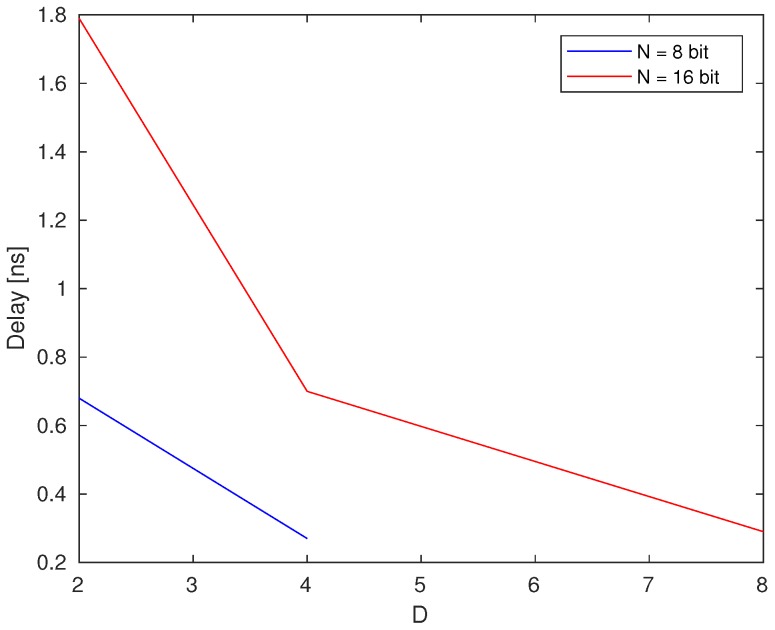
Relation between number of segments in the counter and resulting delay.

An interesting point is the relation between the number of the segments and the resulting delay. An analysis was carried out with 8 bit and 16 bit input data size (Figure 6). As it shows the delay reduces considerably with a bigger amount of segments. Indeed, the architecture under consideration was synthesized with a value D of 8 to achieve best speed.

One of the main goal this paper aimed to fulfill is the high level of concurrency. This was accomplished thanks to the internal structure of the array, distributed on banks which are capable of working both independently and with each other, providing flexibility in the position of the operands that are called to act in the query. To execute a simple query only one cycle is required. Thanks to the modular structure of the array, the maximum throughput achievable working in parallel in PIM multiple banks mode is:

$$throughput_{max_{simple}}=f_{CLK}\cdot N_{ops}.$$

As for composed query two cycles are required to complete the operations. The resulting maximum throughput operating in PIM multiple composed mode is:

$$throughput_{max_{composed}}=\frac{f_{CLK}}{2}\cdot N_{ops}.$$

So, assuming to execute a different query in each of the 16 available banks, we will reach a maximum throughput of 2.45 Gop/s and 9.2 Gop/s for 45 nm and 28 nm respectively. The performance of the proposed PIM architecture was compared with results of other in-memory proposals found in Reference [29] (Table 3).

Noticeably, operations in the proposed PIM array take less clock time compared to other solutions. Moreover, it should be taken into consideration that executing multiple parallel operations would not change the number of clock cycles required. This shows how the throughput mentioned above is obtained. Thus, the maximum degree of parallelism achievable is correspondent to the number of the available banks. Moreover, it is possible to scale the architecture to bigger dimensions as it was conceived as modular, meaning it can be composed with as many banks as wanted. Another possibility is to develop a 3D structure in order to enhance performance. Nonetheless, it would be easy to modify the architecture to make it fit for other types of operations. These results, coupled with the flexibility of the architecture, highlight the potential of the proposed architecture. 

## Figures and Tables

**Table 1:** Synthesis of the fundamental element.

	Memory	Logic	Cell
Non-Combinational Area [mm${}^{2}$]	9.31	2.12	11.43
Combinational Area [mm${}^{2}$]	5.32	15.43	20.75
Total Area [mm${}^{2}$]			32.18
Delay [ns]			0.45

**Table 2:** Synthesis results for 45 nm and 28 nm CMOS technologies.

Parameter	Value (45 nm)	Value (28 nm)
Total area [mm${}^{2}$]	2.33	1.058
$f_{CLK}$ [MHz]	153.4	574.7
Total Power [mW]	49.7	14.07

**Table 3:** Clock cycles comparison for a single query execution.

	f=A·B	$f=A\cdot (\bar{B}\cdot C)$
**Pinatubo [21]**	5	9
**RIMPA [28]**	3	5
**PIMA-Logic [29]**	1	3
**PIM**	1	2
